# On simulating cold-stunned sea turtle strandings on Cape Cod, Massachusetts

**DOI:** 10.1371/journal.pone.0204717

**Published:** 2019-12-04

**Authors:** Xiaojian Liu, James Manning, Robert Prescott, Felicia Page, Huimin Zou, Mark Faherty

**Affiliations:** 1 School of Remote Sensing and Information Engineering, Wuhan University, Wuhan, China; 2 National Oceanic Atmospheric Administration’s Northeast Fisheries Science Center, Woods Hole, Massachusetts, United States of America; 3 Massachusetts Audubon’s Wellfleet Bay Wildlife Sanctuary, Wellfleet, Massachusetts, United States of America; 4 University of Rhode Island, Kingston, Rhode Island, United States of America; 5 Shandong Agricultural University, Tai’an, China; Woods Hole Oceanographic Institution, UNITED STATES

## Abstract

Kemp's ridley sea turtles were on the verge of extinction in the 1960s. While these sea turtles have slowly recovered, they are still critically endangered. In the last few years, the number of strandings on the beaches of Cape Cod, Massachusetts has increased by nearly an order of magnitude relative to preceding decades. This study uses a combination of ocean observations and a well-respected ocean model to investigate the causes and transport of cold-stunned sea turtles in Cape Cod Bay. After validating the model using satellite-tracked drifters and local temperature moorings, ocean currents were examined in Cape Cod Bay in an attempt to explain stranding locations as observed by volunteers and, for some years, backtracking was conducted to examine the potential source regions. The general finding of this study is that sub 10.5°C water temperatures in combination with persistently strong wind stress (>0.4 Pa), results in increased strandings along particular sections of the coast and are dependent on the wind direction. However, it is still uncertain where in the water column the majority of cold-stunned turtles reside and, if many of them are on the surface, considerable work will need to be done to incorporate the direct effects of wind and waves on the advective processes.

## Introduction

Kemp's ridley sea turtles are the smallest and the most endangered sea turtle species in the world and shows little sign of recovery [1,2,3]. Their perilous situation is attributed primarily to the over-harvesting the turtles, their eggs, and incidental capture in shrimping trawls during the last century, causing a ~99% decline in nest production by 1985, based on historical records [3,4,5,6]. In 1970, Kemp’s ridley sea turtles were listed by the U.S. Fish and Wildlife Service [7] as “endangered throughout its range” and have since received Federal protection under the U.S. Endangered Species Act of 1973 [2]. In 1978, a bi-national, multi-agency partnership began working toward establishing the Kemp’s Ridley Sea Turtle Restoration and Enhancement Program, which is still in effect today [6,8,9].

Adult Kemp's ridley turtles are mainly active in coastal waters that are less than 165 feet (<50.3 m) deep, primarily in the Gulf of Mexico, and satellite telemetry has been used over the past few decades to understand migratory patterns and habitat use [4,10,11,12,13,14]. Juvenile Kemp's ridleys are found in shallow waters, often foraging in less than 3 feet (<1 m) of water [5]. Juveniles are also found in the Gulf of Mexico [15,16] as well as along the eastern seaboard of the United States and as far north as Nova Scotia, Canada [17] leaving warmer waters to feed on the abundant crabs and other prey found in the colder northern waters [18].

Sea turtles foraging along the northeast coast of the United States migrate south to warmer waters to overwinter. Hart et al. [19] discuss the seasonal variability of both loggerhead and Kemp’s ridley strandings based on drifter bottles and particle tracking. As in the loggerhead study by Santos et al. [20], the objective is often to document the location and probable cause of mortality [21] using a variety of methods such as the deployment of coded oranges [22]. In many cases, it is abrupt changes in sea surface temperature (SST), typically associated with storm surges and cold fronts, that trigger cold stunning. Turtles in the vicinity of Virginia, tend to migrate south in late fall each year [23,24], but, even in Florida, significant mortalities have been reported [25] as a result of being trapped in shallow, water bodies, such as estuaries.

In laboratory experiments, Schwartz [26] demonstrated that Kemp's ridleys become inactive when temperatures drop below 10°C because hypothermia begins to affect physiological processes. However, the results of the experiment also showed a higher resistance in Kemp’s ridleys than in other sea turtle species [26]. If a sea turtle becomes hypothermic, hereafter referred to as “cold-stunned”, there is little chance of survival without assistance. Morreale et al. [27] studied cold-stunned stranding events in the late 1980s on Long Island, NY. Burke et al. [28] specifically addressed the causes of these events and found one year, in particular, when fewer strandings occurred. They attributed this inter-annual variability to slight changes in the prevalent wind direction at the critical time of year when the water temperature fell below 10°C.

When Kemp's ridleys north of Cape Cod Bay delay their southern migration to warmer waters in early fall, they are likely to become trapped in the region for the duration of the season. The oceanographic conditions in this area are highly variable, dependent on source waters arriving from the north (Maine Coastal Current) that occasionally injects water into Massachusetts Bay and its subregion, Cape Cod Bay as well as episodic storm events that pass by the area and stir up an otherwise stratified water mass. When these events suddenly occur in late fall, sea turtles are thought to be trapped in Cape Cod Bay due to the presence of the Cape Cod landmass and the hook-like structure of the peninsula, resulting in cold stunning once water temperatures drop below 10.5°C [29]. These turtles, typically 2 to 3 years old, are sized at 26.9 cm mean straight carapace length [29], and similar to the size of a dinner plate or a serving platter [30]. Their small size makes it difficult to attach satellite telemetry devices, so these juvenile Kemp’s ridleys are more difficult to track than the larger turtles being tracked in the Gulf of Mexico [15,16,31]. In recent years, the number of sea turtles stranding on Cape Cod beaches has increased, with a record number or 1200 stranded turtles recovered during the 2014 cold stunning season. During the summer months there are a few strandings reported because of turtles getting a) entangled in fishing gear, b) struck by boats, or c) stranded on exposed sandbars during the ebb tide. However, these numbers are minimal compared to the number of sea turtle that strand in Cape Cod as a result of cold stunning. Cold-stunned turtles are extremely weak, often immobile, and, in many cases, wash ashore. Once stranded, cold-stunned turtles need to be rescued by the dozens of trained volunteers each fall. Since 1979, in connection with the Sea Turtle Stranding and Salvage Network, Massachusetts Audubon’s Wellfleet Bay Wildlife Sanctuary (Wellfleet, MA) staff and volunteers have patrolled Cape Cod beaches, on the lookout for cold-stunned turtles. Once recovered, the turtles are rapidly transported to the New England Aquarium (Boston, MA) to begin evaluation and rehabilitation. Over the last decade, the number of cold-stunned strandings has increased by nearly an order of magnitude relative to the strandings in the preceding decades.

This paper explores the transport of cold-stunned Kemp's ridleys using the Finite Volume Community Ocean Model (FVCOM), a numerical ocean model well-suited to simulating coastal ocean processes [32]. After validating the FVCOM using drifter data and mooring data (see Supplementary Material Appendices, hereafter SM-I, SM-II, or SM-III), we looked at the number of sea turtles stranded in various towns along Cape Cod beaches from 2012 to 2015 in an attempt to explain these distributions based on the FVCOM-derived simulations, particle tracks, drifter tracks, and observations of water temperature.

## Methods

The study was carried out in Cape Cod Bay. No animals were involved with the sampling, so no specific permissions were required for these locations/activities. While dozens of drifters were deployed in the bay, nearly all were recovered ashore—only one escaped from the bay and was lost at sea.

We use a combination of observations and model output to examine the flow fields in Cape Cod Bay. While we have dozens of drifter tracks collected within the bay, it is certainly not enough to describe the very complicated circulation due to a dynamic combination of tidal, thermohaline, and wind-driven processes. Since drifters and moorings were not always deployed at all times during the turtle stranding seasons, we use the model to help resolve both the spatial and temporal variability. We use the observations primarily to help validate these model simulations as described in more detail in SM-I. In the sections that follow, we describe the a) model, b) drifter and temperature observations, c) our method to simulate drifters, d) our method to bin both observed and modeled fields, and e) the turtle stranding database.

### FVCOM

The FVCOM is a prognostic, unstructured-grid, Finite-Volume, free-surface, three-dimensional (3-D) primitive equations Community Ocean Model developed originally by Chen et al. [32,33]. Given the topographic complexities of shelf regions such as Cape Cod Bay, the finite volume method on unstructured grids is an ideal means of solving conservation equations in the coastal ocean. More details on the model and a comparison of the output with observations are provided in SM-I.

FVCOM has been used on several grids spanning the Northeast Continental Shelf of the U.S. and is still evolving. The third-generation grid (GOM3) was used in this experiment covering the waters of the Gulf of Maine and Georges Bank and hourly flow fields are available from 1978 to 2015. The GOM3 grid was developed primarily for the prediction of coastal ocean dynamics, with horizontal grid resolution ranging from 20 meters to 15 kilometers. It includes tidal constituents, is forced by a global FVCOM, and assimilates remote observations of sea surface heights and temperatures. Vertical profiles of temperature and salinity when available from both shipboard and moored instruments at sparse locations on the shelf were also assimilated. The vertical domain was divided into 45 s-coordinate levels. We also experimented on a finer-resolution Massachusetts Bay grid, hereafter “MassBay”, that had hourly flow fields available from 2011–2014. In the next few years, model flow fields will be available on the “GOM4” and “GOM5” grids that have nearly double the resolution especially in the coastal regions and span the entire Northeast US shelf region.

### Drifter observations

**Fig 1:**
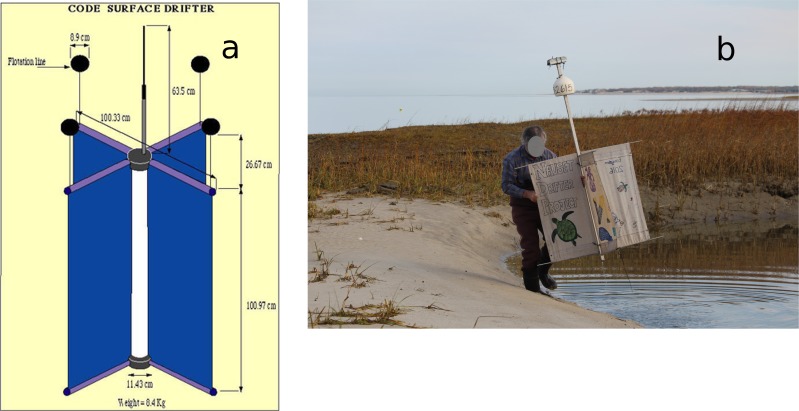
Schematic of standard surface drifter from Poulain [34] (left) and photo of surface drifter used in this report being recovered after stranding in Barnstable MA (right).

The satellite-tracked surface drifters used in this study enabled us to track the currents in the top meter of the water column [34]. This type is typically referred to as the “Davis-style” or “CODE” drifter first developed for the Coastal Ocean Dynamics Experiment in the early 1980s (Fig 1) [35]. Our version of this drifter consists of a metal-frame and four fabric sails measuring 91 cm long and 48 cm wide. The drifters were student-built units deployed primarily by local fishermen in the vicinity of Cape Cod from 1988 to present with tracks archived for public access. More than a thousand of these drifters have been deployed off the New England coast in the past few decades [36,37]. The primary purpose of these drifters is to validate ocean models (see SM-I). A total of 58 tracks within Cape Cod Bay were used in this study (prior to 2017) but there are many more in recent years and in other areas of the Northeast Shelf (see SM-III).

### Moored temperature observations

**Fig 2:**
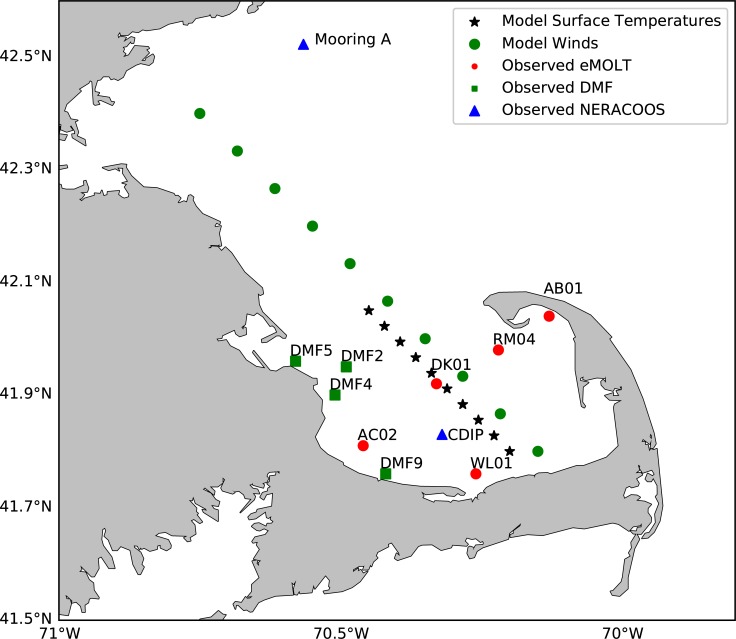
Locations of moored observations used in this report including two NERACOOS moorings (blue triangles), five eMOLT moorings (red circles) and four Massachusetts Division of Marine of Fisheries moorings (green squares). Also posted are the positions where wind and surface temperature were extracted from the models.

There are at least three different sets of moored ocean temperature observations available in the Northeast U.S. The Northeastern Regional Association of Coastal Ocean Observing System (NERACOOS) typically has several mooring sites in the Gulf of Maine [38]. In addition to the long-tern “Mooring A” on the northern boundary of Mass Bay, mooring “CDIP” was added in 2017 within Cape Cod Bay as shown in Fig 2. The Environmental Monitors on Lobster Trap Project (eMOLT) [39] comprises another collection of moorings with almost 20 years of hourly bottom temperatures from dozens of locations off the New England coast including several within Cape Cod Bay. Finally, the Mass Division of Marine Fisheries (DMF) maintains several diver-serviced bottom temperature time series at a set of shipwrecks off the Massachusetts coast. The combination of these three datasets typically provides time series data at several sites near and within Cape Cod Bay through most of the year.

### Sea-surface temperature observations

In addition to the modeled sea-surface temperature (SST) estimates that are available hourly, we also have obtained observations of SST from satellite. While clear images are only available a few times per month in this region, they provide another snapshot realization of the conditions in Cape Cod Bay. For this report, we accessed the raw data prepared by the University of Delaware and posted via the MARACOOS.org website just to examine a few days before and after a large stranding event in 2014.

### Simulating forward and backward turtle trajectories

The science of tracking particles through the coastal ocean is still evolving [33] and there are a variety of techniques [29,37,38] that can be applied depending on the physical and biological behavior of the particle or animal under investigation. In this case, since the animal’s position in the water column and its ability to regulate that depth is unknown, we present here a very simple case of moving it through the model’s surface fields using the nearest grid nodes with hourly time steps with no consideration of other factors such as wind and waves. The comparison of observed and modeled particle tracks is presented in SM-I where we calculate the “separation distance”, for example, between the observed and model trajectory.

For the case of backtracking the cold-stunned turtles to estimate their origin, we first needed to get the simulated turtle off the beach to begin moving it backward through the water. The process of selecting a “short distance off the beach” is not easy. We experimented with multiple methods to solve this problem (SM-II). For study, we chose the method which involved a) finding the nearest point on the model-based coastline to the stranding location, b) calculating the line from the specified stranding to that point, and then c) extending the line ***X*** km out to sea. More complex methods, as described in SM-II, seemed to be more sensitive to the resolution of the coastline. After experimenting with several values, we chose to set ***X*** = 1.5 km. As suggested in Nero et al. [31], the value selected is partly a function of the bathymetric slope offshore wherein their case ***X*** varied from 0.3 km to 1.0 km. The backtracking is terminated when the estimated water temperature exceeds the cold-stunned level. For the reasons discussed below, we chose this to be 10.5 ^0^C. We backtracked turtles for 2012 and 2013. We selected 167 turtle strandings in those years where the simulated trajectory stayed off of the beach. In other words, we focus on only the 167 cases where the backtracking generated origins within the bay.

Note: Since there were evidently issues with 2014 model hindcast associated with boundary forcings and assimilations, we chose to focus only on the previous two years in this analysis.

### Binned averaged current and wind

To examine the surface current conditions in the bay for a specified period of time, we bin-averaged both observed and modeled flow fields in on 0.05° (~5km) bins. The “observed current” is derived from averaging the first differences of the near-hourly drifter fixes within each bin. The model data is the hourly output of the FVCOM as described above. We averaged the “observed current”, “modeled current”, and the “modeled wind stress”. The modeled current in each grid cell is the average velocity of all nodes within each bin whenever drifters were present in the bin. In reality, there are different types of current velocity (Lagrangian vs Eulerian) and technically, we cannot assume that they are identical. Nevertheless, after removing the tidal effects by sequentially averaging over the solar (24 hours) and lunar (24.841 hours) periods, we calculated the average surface current of each bin. The wind stress was derived from the same weather model that drives the FVCOM ocean model, which is a local implementation of the NCAR Mesoscale Model [40].

### Turtle stranding data

Massachusetts Audubon’s Wellfleet Bay Wildlife Sanctuary provided the sea turtle stranding data used in this experiment (unpublished data). These data include information on date and location of stranding for each cold-stunned sea turtle from 1991 to 2015. Hundreds of trained volunteers have been participating in this project since 1979. Volunteers walk Cape Cod beaches each fall in search of stranded sea turtles to initiate the rescue and recovery process. To quantify the relationship between wind stress (derived from the MM5 model noted above) and the number of strandings, we calculated the correlation between these two variables when the temperature dropped below 10.5°C. Since it is not only the strength of the wind that is important but also the persistence of the wind over time, we chose to calculate the “3-day sum of the wind stress components”.

## Results

### Kemp's ridley strandings on Cape Cod

**Fig 3:**
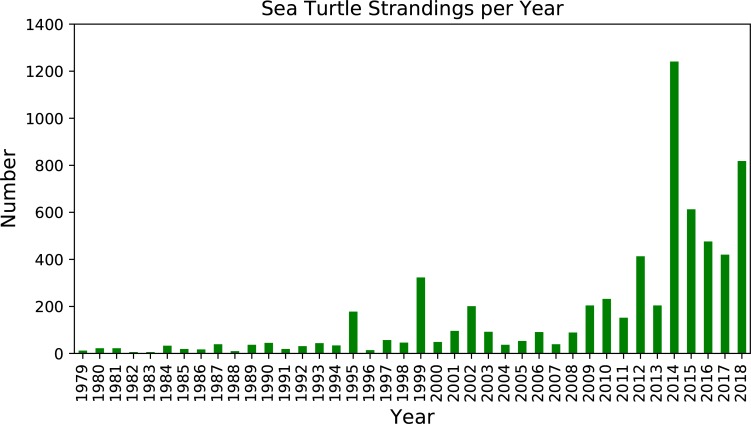
1979–2018 Cape Cod Bay turtle strandings.

Fig 3 displays the number of Kemp's ridleys stranded on Cape Cod beaches from 1979 to 2018. As shown, the numbers of strandings varies from year to year but have increased dramatically in recent years. Although records of earlier stranding numbers have little information on the methods used to collect data and documented search efforts, search efforts and data collection have been consistent since the early 1990s (R. Prescott, personal communication).

**Fig 4:**
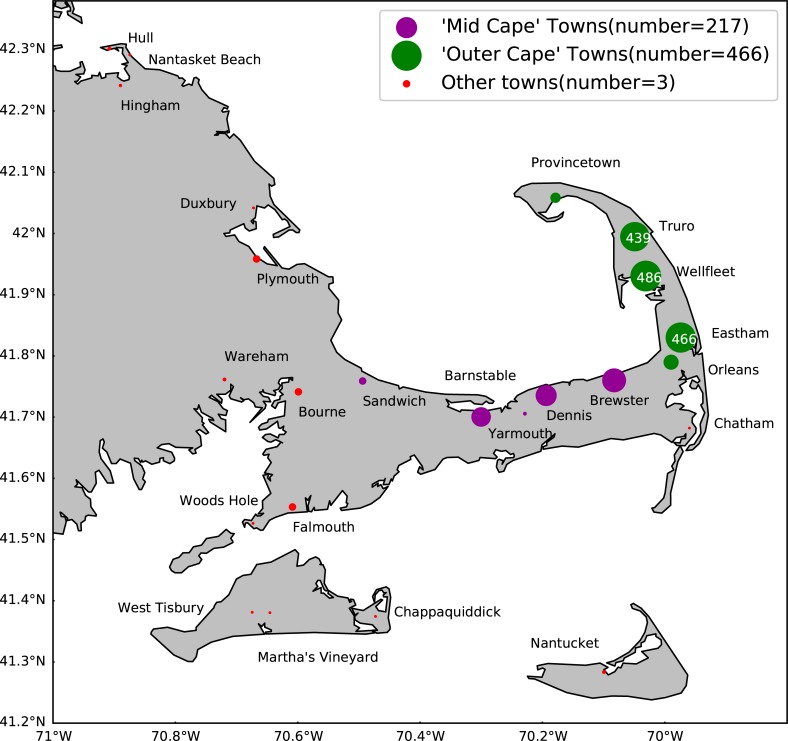
Total turtle strandings per town from 2012–2015. Most of the Outer Cape towns (green dots) had >400 strandings while some of the Mid Cape towns (purple) had ~200. All strandings from these sections were found on the Cape Cod Bay side of the towns.

**Fig 5:**
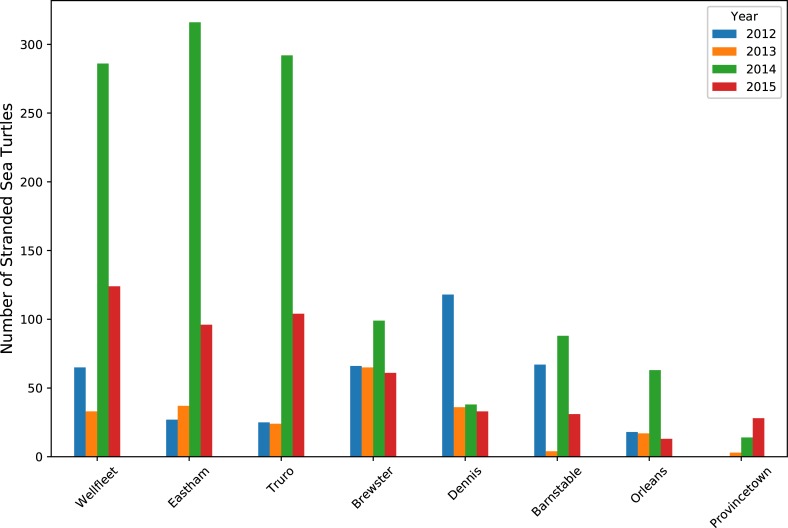
Chart of the number of strandings by town between 2012–2015 showing most in the first three Outer Cape towns.

Turtles were recovered in several towns each year between 2012 and 2015 (Figs 4 and 5). The towns with the largest numbers of strandings between 2012–2015 were located on the Mid to Outer Cape (the land farthest east). Among these towns, each reported strandings in every year of the study period. We hypothesize that year-to-year changes in stranding locations are primarily due to small changes in both the speed and direction of subtidal surface currents.

### Examining binned current and wind

**Fig 6:**
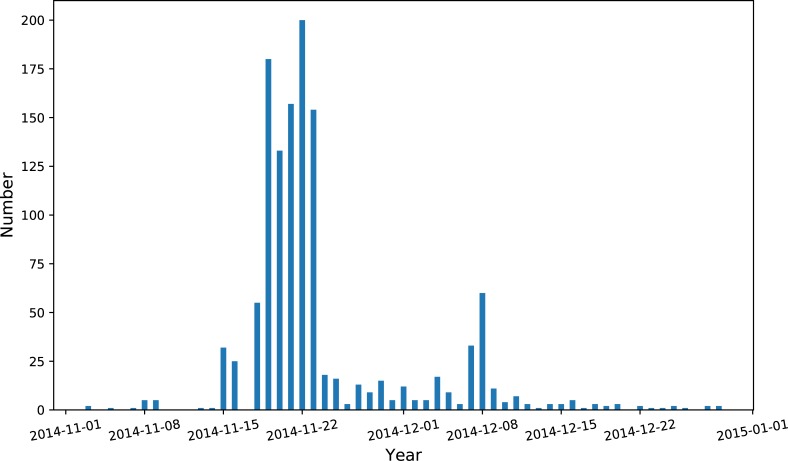
The number of turtles stranded on Cape Cod Bay beaches in Nov-Dec 2014.

**Fig 7:**
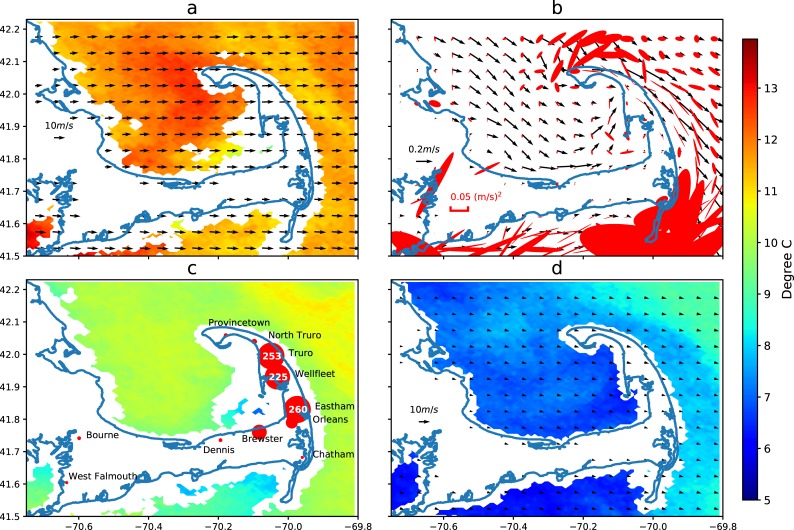
November 18–23, 2014 mean wind (black arrows) with November 3, 2014 SST (a), MassBay grid simulation of mean ocean surface currents (black arrows) during that time overlaid on surface current variance ellipses (pink) (b), and distribution of turtle strandings by town with November 19, 2014 SST (c), and mean wind for the entire month of November 2014 with December 26, 2014 SST (d). The legends are posted on the landmass on the western side of the charts. The largest red dot in panel c denotes 260 turtles stranded in Eastham during the period 18–23 November 2014.

Despite recent efforts to survey the bay from both ships and planes, we do not know the exact location of the cold-stunned turtles in the bay or the water column. However, considering cold-stunned turtles have little, if any, mobility, we assume they generally follow the surface currents with some effects by the wind and waves. It is expected that more proficient predictions of stranding positions may be made using observed ocean surface currents and wind stress to numerically advect water parcels forward. Fig 6 shows the number of turtles stranded on Cape Cod beaches per day in late 2014, with most occurring the week of November 21st. Before examining the hour-by-hour time series of events in subsequent figures, Fig 7 maps the mean current and the mean wind stress from the entire period from November 18–23, 2014, and the distribution of the stranded sea turtles for these six days.

**Fig 8:**
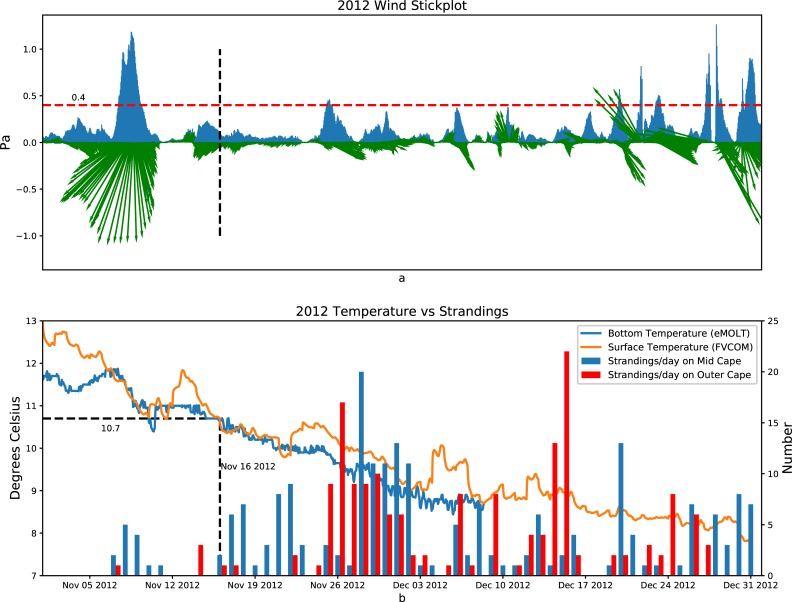
2012: Top panel shows wind stress (Pa) direction shown in green vectors (north up) and magnitude in blue. The dashed red line represents the critical wind stress level of 0.4 Pa. Bottom panel shows Mid Cape strandings (blue bars) the Outer Cape strandings (red bars), modeled surface temperature (orange line), and observed bottom temperature (blue line) in 2012.

**Fig 9:**
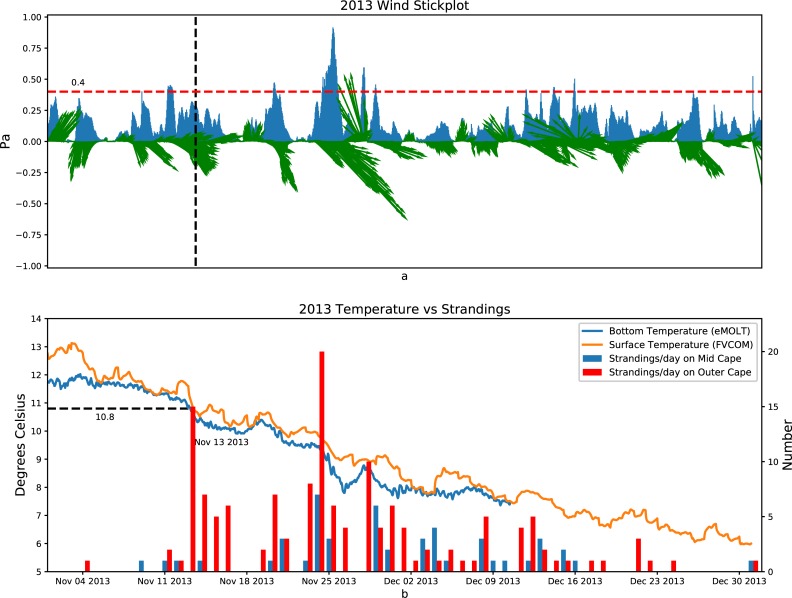
2013: Top panel: Wind stress with direction shown in green vectors (north up) and magnitude in blue. Bottom panel: Mid Cape (blue bars) and Outer Cape (red bars) strandings, modeled surface temperature (orange line), and observed bottom temperature (blue line) in 2013.

**Fig 10:**
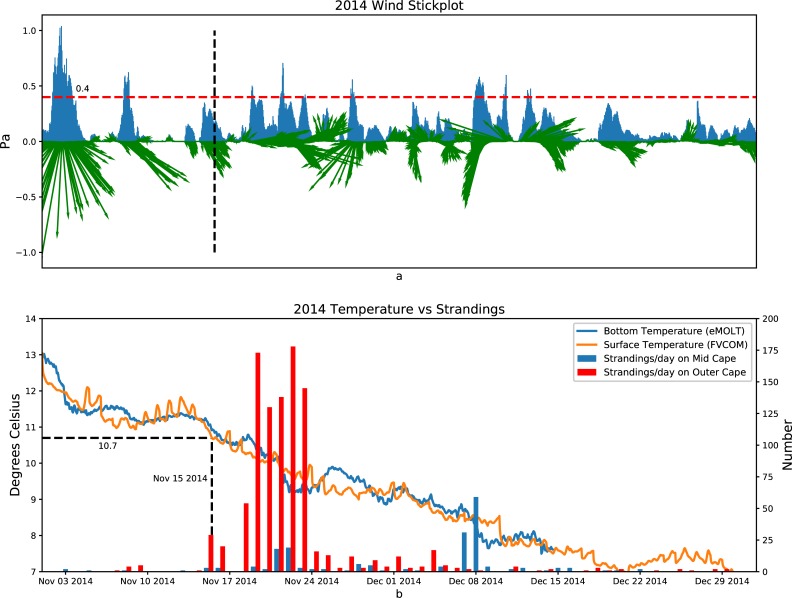
2014: Top panel: Wind stress with direction shown in green vectors (north up) and magnitude in blue. Bottom panel: Mid Cape strandings (blue bars), Outer Cape strandings (red bars), modeled surface temperature (orange line), and observed bottom temperature (blue line) in 2014.

From November 18–23, 2014, as depicted on the turtle stranding map (Fig 7C), Truro, Wellfleet, and Eastham had the most strandings (253, 225, and 260, respectively), and their positions are consistent with the oceanic currents and winds shown by the simulated current and wind maps (Fig 7A and 7B), distributed in the area where currents and wind stress are most intense, and in their direction. It can be seen that the combination of ocean current and wind calculated by the FVCOM can be used to explain the general distribution of strandings. Comparing the mean wind for one week in mid-November 2014 (Fig 7A) to the mean wind for the entire month of November 2014 (Fig 7D), we see the intensity of the mid-November period explains the unusually high rate of strandings on the Outer Cape. The intensity of these events is best described in the form of the time series plots below (Figs 8–10). Future turtle rescue efforts can use the FVCOM 3-day forecast model to simulate stranding events and help volunteers focus their search efforts.

As previous studies have found, when the water temperature of the Cape Cod Bay is less than 10°C, the physiological processes of Kemp's ridley turtles will slow to the point that their ability to actively swim is affected and they are at the mercy of the wind, waves, and currents [29]. As shown in Fig 2, we set up 10 temperature detection points and 10 wind stress monitoring points across Cape Cod Bay and Massachusetts Bay to extract the temperature and wind stress simulated by the model. In the following plots (Figs 8–10), we examine the relationship between wind stress and strandings especially when the ocean temperature is below 10.5°C.

Figs 8–10 show the relationship between the wind stress (top panels), and the number of strandings in the Outer Cape and the Mid Cape (bottom panels) towns in 2012, 2013, and 2014, respectively. The average of the surface temperature simulated by all the FVCOM points (specific model sites shown in Fig 2) and the average of the bottom temperature observed in Cape Cod Bay (specific observation sites shown in Fig 2) are also plotted in the bottom panels. The wind stress vector is the average simulated by the FVCOM points (sites shown in Fig 2). When the temperature is less than 10.5°C and the wind stress from a particular direction is greater than ~0.4 Pa, the strandings generally increase but both conditions must be met. In the case of 2012 (Fig 8), for example, the strong wind in early November resulted in near-zero strandings since the water temperature was still above 11°C. In the case of 2013 (Fig 9), there was a pair of events with strong northwesterly wind at the end of November that coincided with a drop in temperatures resulting in peaks in strandings around 26 November and 1 December. Note the strong winds (>0.4 Pa) in mid-December 2013 resulted in very few strandings presumably due to the turtles having fled or already stranded by that time. In the case of 2014 (Fig 10), the wind in mid-to-late November was not strong but it was persistently from the west over many days with a drop in both surface and bottom temperature of a few degrees, which resulted in a historically high number of strandings.

**Fig 11:**
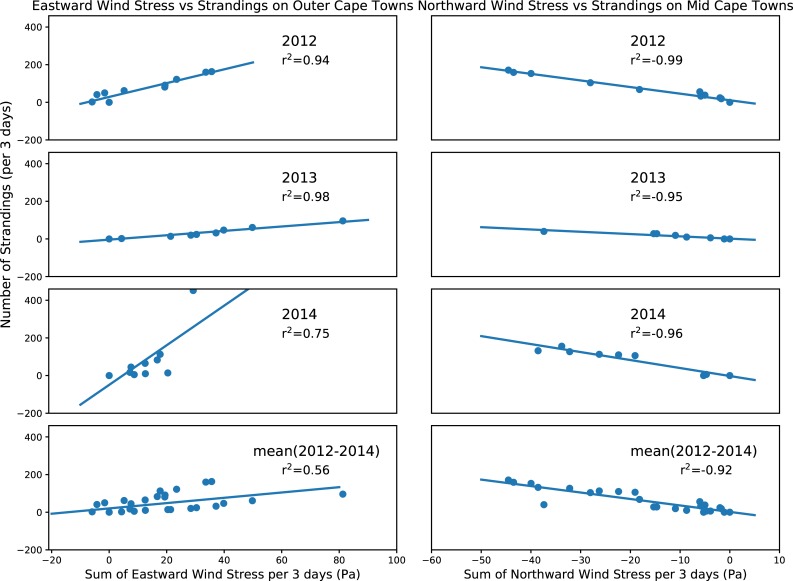
Linear correlations between eastward (left) and southward (right) components of the wind stress and number of strandings (summed over 3 days) in 2012, 2013, 2014 and mean (2012–2014) for Outer Cape (left) and Mid Cape (right) towns.

Fig 11 (left-hand panels) shows the correlation between the number of turtles stranding in Outer Cape towns and the 3-day sum of the **eastward** wind stress in 2012, 2013, 2014 and mean respectively. Fig 11 (right-hand panels) shows the correlation between the number of turtles strandings in Mid Cape towns and the **southward** wind stress in 2012, 2013, 2014, and mean respectively. In most cases, the correlation is greater than 90% and exceeds 95% in several cases. Wind stress and the number of strandings per 3 days show a linear correlation.

**Fig 12:**
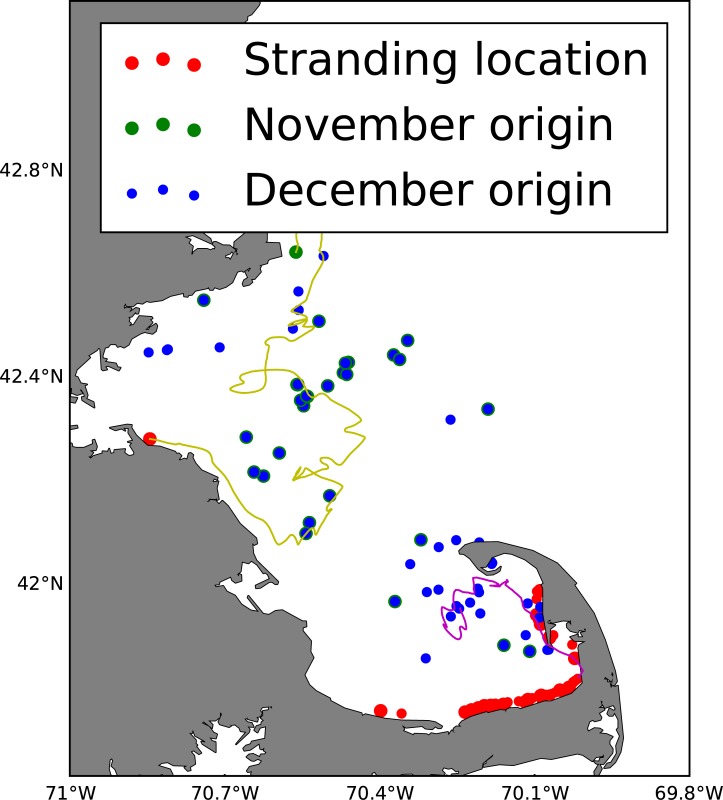
Backtracked positions of stranded sea turtles in 2012 and 2013. Red dots show the landing locations in November and December. Blue and green dots show estimated locations of backtracked turtles that stranded in November and December, respectively, when they first encountered <10.5°C water. These estimates assume that passive (non-swimming) turtles were only transported by currents when the modeled surface temperature was less than 10.5°C. The yellow and magenta lines are examples of backtracking turtles that stranded in the western-most and eastern-most locations, respectively. According to the model, these turtles were first cold-stunned off Gloucester, MA and mid-Cape Cod Bay, respectively.

**Fig 13:**
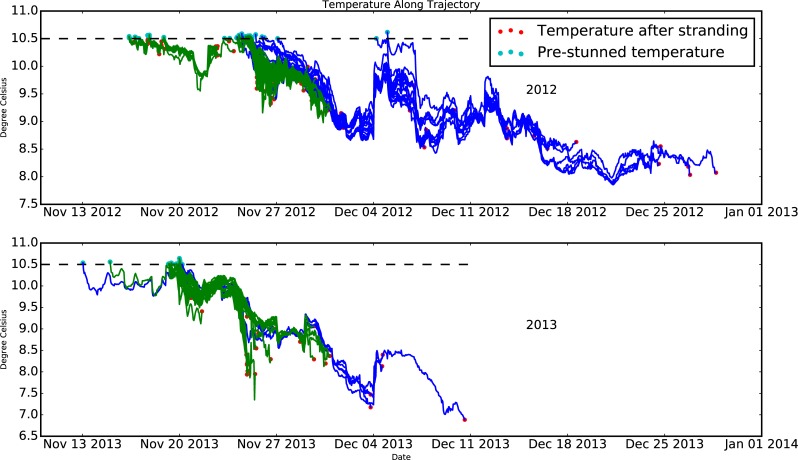
Modelled temperature of backtracked surface particles in 2012 (top panel) and 2013 (bottom panel).

Finally, to estimate the source of turtles (i.e. their distribution/origin at the time of first being cold-stunned), we look at the backtracking results (Fig 12). We can also look at the estimate of temperature along their track (Fig 13). In these time series we see that a) many of the stranded turtles were exposed to <10.5°C water in mid-to-late November and b) there were more December stranding events in 2012.

One of the prominent questions driving this research is the location of the sea turtles when they were first exposed to the <10.5°C temperature that resulted in their November stranding. Our results show that turtles stranded in November 2012 and 2013 may be cold-stunned at a variety of locations (blue dots in Fig 12) primarily in Cape Cod Bay but some in Massachusetts Bay. Turtles stranded in December 2012 and 2013 (green dots in Fig 12) may originate even further away, some as far as northern Massachusetts Bay. After first being exposed to <10.5°C surface waters, the turtles are in temperatures that vary considerably but gradually decrease by a few degrees at most before stranding (Fig 13). While there is a great deal of uncertainty in these model-derived particle tracks as quantified in SM-I, these results provide some insight into the potential source region of cold-stunned turtles.

## Discussion

Although state-of-the-art coastal ocean models like the FVCOM can help simulate cold-stunned turtle pathways, there are a variety of issues to consider. First, there are uncertainties in the numerically simulated drifter track results (Fig 12), with differences of up to several kilometers per day relative to the observed satellite-tracked drifter tracks. These uncertainties stem from the assumption that cold-stunned sea turtles are completely passive (i.e. no active swimming occurs) and that there are no differences between individuals (e.g. different thermal inertia or tolerances). The 200-meter-per-kilometer difference in modeled vs observed trajectory (as shown in SM-I) need to be improved considerably before these particle tracking tools can be used confidently and operationally. Simulated trajectories will also differ from actual trajectories due to wind and waves influencing cold-stunned turtle trajectories. There is a growing body of literature on surface wave effects on the transport of surface particles. Much of this research comes from the U.S. Coast Guard search and rescue operations [41,42] as well as recent studies on oil spill dispersion. Methods have been devised to add the effects of multiples forces including direct wind effect, breaking waves [43], Stokes drift [44], inertial effects [45], and these will need to be considered in future work. Since it is still uncertain where in the water column these cold-stunned turtles reside, we chose to focus on surface current fields as a first approximation in this study but these results would change significantly for turtles floating directly on the water surface.

Additional uncertainties are introduced with the tracking methodology near the boundary of the model grid (i.e. in shallow waters near the shoreline). We need to further explore algorithms for forward and backward particle tracking near the coast especially in this study area where there is a large expanse of wetting and drying tidal flats and a tidal range near 30 cm. The primary goal of future work will be to improve our particle tracking models, which will include effects of wind, waves, and processes near the coast.

**Fig 14:**
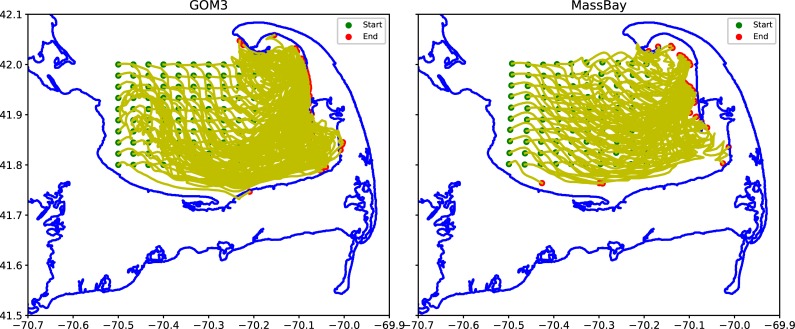
Comparing particle tracks on the GOM3 (left) and MassBay (right) grid during the week of 18–25 Nov 2014.

Despite these limitations, models like the FVCOM are improving every year particularly with increasing grid resolution, incorporation of more realistic bathymetry, and more observations to assimilate. To examine the effect of different grid resolution, for example, we conducted a sensitivity study comparing particle tracks on the “GOM3” vs “MassBay” grid (Fig 14). These results show that the particles released on the GOM3 grid stranded on the Cape Cod beaches faster than those released on the MassBay. While the final stranding locations would likely be similar, the particles on the latter grid were not yet stranded at the end of the week-long simulation.

More observations are needed to monitor in-situ conditions throughout Cape Cod Bay including more realizations of the deeper currents not depicted by the standard surface drifters used here. There is uncertainty in both the deeper currents and the depth at which the turtles generally reside during this critical time in the fall. If we know where these turtles originate and at what depth of the water column, divers or ROVs (remotely operated underwater vehicles) can be deployed to help locate the turtles during the cold stunning season before stranding. There is also a chance that some turtles may be on the bottom waiting for the water to warm. Very few have been observed near the surface whenever searches have been conducted.

Research has shown that the frequency and severity of extreme weather events are increasing [46,47] and therefore could affect the number of sea turtles impacted by cold stunning conditions. As conservation efforts work to increase sea turtle populations [3], as more individuals start their migration from the Gulf of Mexico beaches, and as more of these tropical species migrate north with warming conditions [48], will we see a larger sea turtle population in Cape Cod Bay in the future, which may result in larger stranding numbers.

Despite all these limitations associated with our approach, we have been able to combine a set of observations and model results to describe the general processes governing the stranding events. When persistent strong winds from the north and west move the surface layers shoreward, the water column mixes to some extent and the water temperature goes below a critical level (10.5°C), peak strandings occur in particular towns depending on the wind direction. As the stranding database continues to grow in subsequent years and more drifters are deployed (see SM-III), the same methods described here can be applied to improve the certainty of our results.

## Conclusion

With some specified degree of uncertainty, the ocean current and temperatures simulated by the FVCOM ocean model are similar to that observed by satellite-tracked drifters and moored observations. This model can help predict the stranding locations of Kemp's ridley sea turtles on Cape Cod beaches and also be used to backtrack their source. In the forward prediction modeling, the FVCOM helped explain, for example, the anomalously large stranding event that occurred on the Outer Cape Cod between November 18–23, 2014. In general, the geographic distribution of standings is consistent with the model simulation of ocean currents and winds. Given these findings, coastal ocean models such as the FVCOM can shed light on potential stranding locations and may guide search and rescue efforts in the future.

Backtracking experiments/scenarios with the FVCOM were used to estimate the origin of stranded Kemp's ridley turtles in 2012 and 2013, showing with a specified degree of uncertainty that most turtles stranded in November were originally located in a variety of locations in both Cape Cod Bay and Massachusetts Bay when they were first exposed to cold-stun temperatures (<10.5°C). The turtles stranded in December may have originated well outside of Cape Cod Bay just a few weeks earlier.

There are still many uncertainties as to when the turtles are cold-stunned but a simple regression of different components of the wind stress (with water temperatures below the 10.5°C threshold) vs the number of strandings indicate that values greater than 0.4 Pa will induce more stranding events (r^2^ = 95). As expected, strong winds out of the west will result in Outer Cape strandings and strong winds out of the north will result in Mid Cape strandings.

While progress has been made in simulating the stranding events, considerably more work needs to be conducted to a) determine the depth of the water column occupied by the turtles and b) the differential effects of surface advection due to wind and waves relative to near-surface currents.

## Supporting information

S1 Supplementary material CCBay manuscript More on circulation model and the validation of model results. This file includes five sections 1) description of the numerical model, 2) bin-averaged current and wind, 3) forward tracks through modeled flow compared with drifter tracks, 4) modeled vs observed temperatures, and 5) more references.
It contains seven figures.
(PDF)

S2 Supplementary material CCBay manuscript On methods to get turtles off the beach for particle tracking. This file has a description of how we project the beached turtle location a set distance off the coast in order to get it started in the particle backtracking routines. It includes three figures to illustrate the process along with a link to the Python code.
(PDF)

S3 Supplementary material CCBay manuscript More on drifters. This file includes two sections on 1) animating tracks including links to the code and example animations and 2) a description of the drifter archive including three figures with some details on the years and months drifter tracks are available for Cape Cod Bay as well as the total on the entire Northeast Shelf.
(PDF)
