# Supplementary material for: On simulating cold-stunned sea turtle strandings on Cape Cod, Massachusetts
Source: PLoS One. 2019 Dec 4;14(12):e0204717. doi: 10.1371/journal.pone.0204717 (PMC6892539; doi:10.1371/journal.pone.0204717)
Supplement: S2 Supplementary material CCBay manuscript — This file has a description of how we project the beached turtle location a set distance off the coast in order to get it started in the particle backtracking routines. It includes three figures to illustrate the process along with a link to the Python code. (PDF) [file pone.0204717.s002.pdf]

Supplementary Material related to:

"On simulating cold stunned turtle strandings on Cape Cod"

Appendix 2:

On methods to get turtles off the beach for particle tracking

As noted in the methods section, a variety of techniques were tested to get the stranded turtles off the beach in order to initiate backward particle tracking. Since most of the stranding reports have positions outside the model grid, we needed to define a new location some distance off the beach before the particle would move in the modelled flow fields. The code we used for this application is available at:

https://github.com/jamespatrickmanning/Lei_et_al_2019_CCBAY/tree/S2_method_off_beach.

The method we actually used is as follows and illustrated in Fig S2_1. The black line indicates the digitized coastline as defined by series of points. The spacing between these points is often different. The red point is the point where the turtle is stranded, the green point is the coastline point closest to the red point, and the blue line is the line between the stranded point and the nearest coast point. The yellow dot is a point where the green dot extends 1.5km along the blue line into the sea.

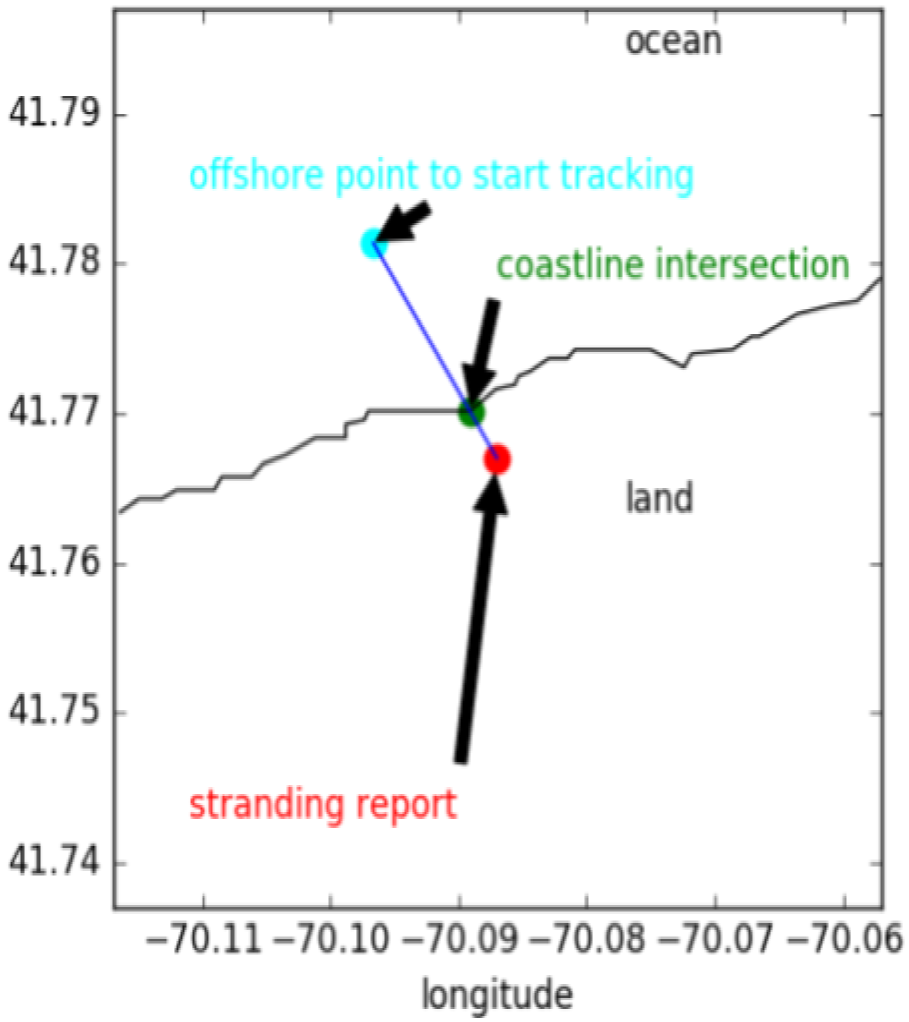

Fig S2_1. Simple method as used in the paper where we locate the nearest point on the coastline (green dot) to the stranding report (red dot) and then extend the line between them to an offshore point (cyan

As shown in Fig S2_2 and S2_3, an alternative method was considered. This method generates a circle around the stranding (red point) with diameter "d=X_1 km" , calculates the two coastline points farthest from the red point in the circle, draws a straight line between them, calculates the

midpoint of the line (green point), connects the red point and the green point, and extends X_2 km to the sea to get the cyan point. We found that the end result of this more complicated method was sensitive to a) the radius of the circle and b) the resolution of the coastline.

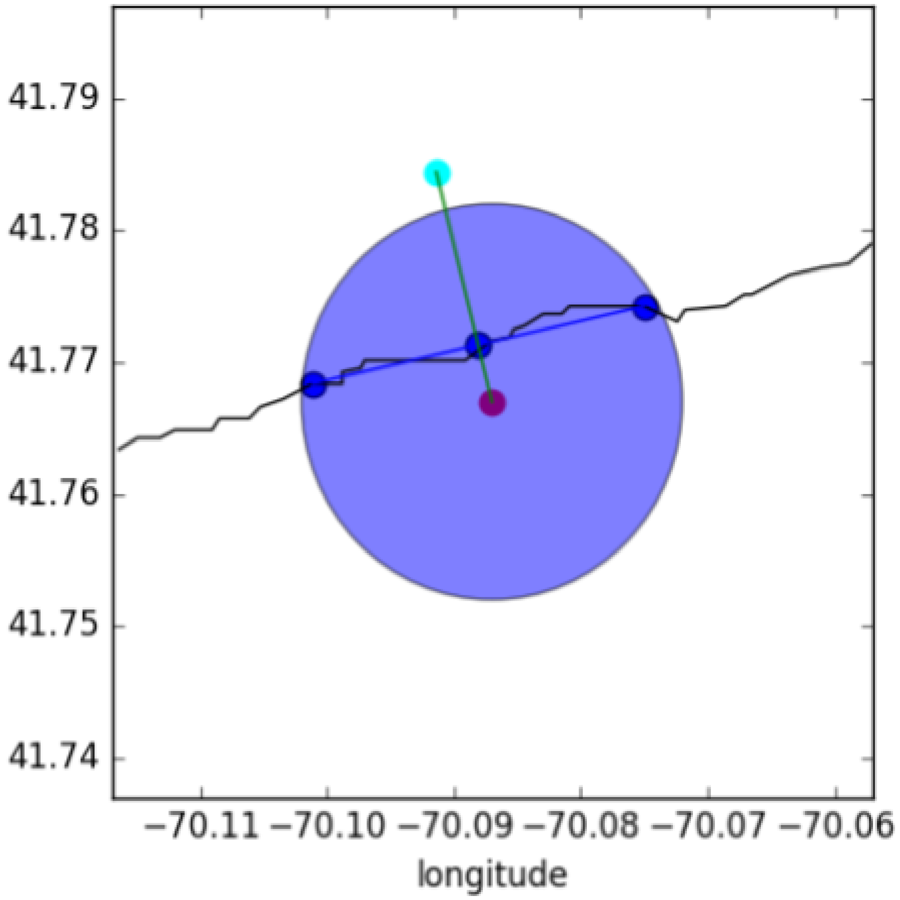

Figure S2_2. More complex method of finding the intersection of a circle of certain radius (in this case 0.015 deg) then drawing a line perpendicular to the line connecting them offshore.

In Fig S2_2, X_1 = 1.7km and X_2 = 1.5km and in Fig. SM2_3, X_1 = 1.1km and X_2 = 1.5km. Due to the different radius of the circles, the yellow position points obtained in Fig SM2_2 and Fig S2_3 are significantly different. In cases of very complex coastline, neither method will provide ideal results for each stranding. As noted in the manuscript, some tracks were abandoned when they failed to advect offshore using the simpler method.

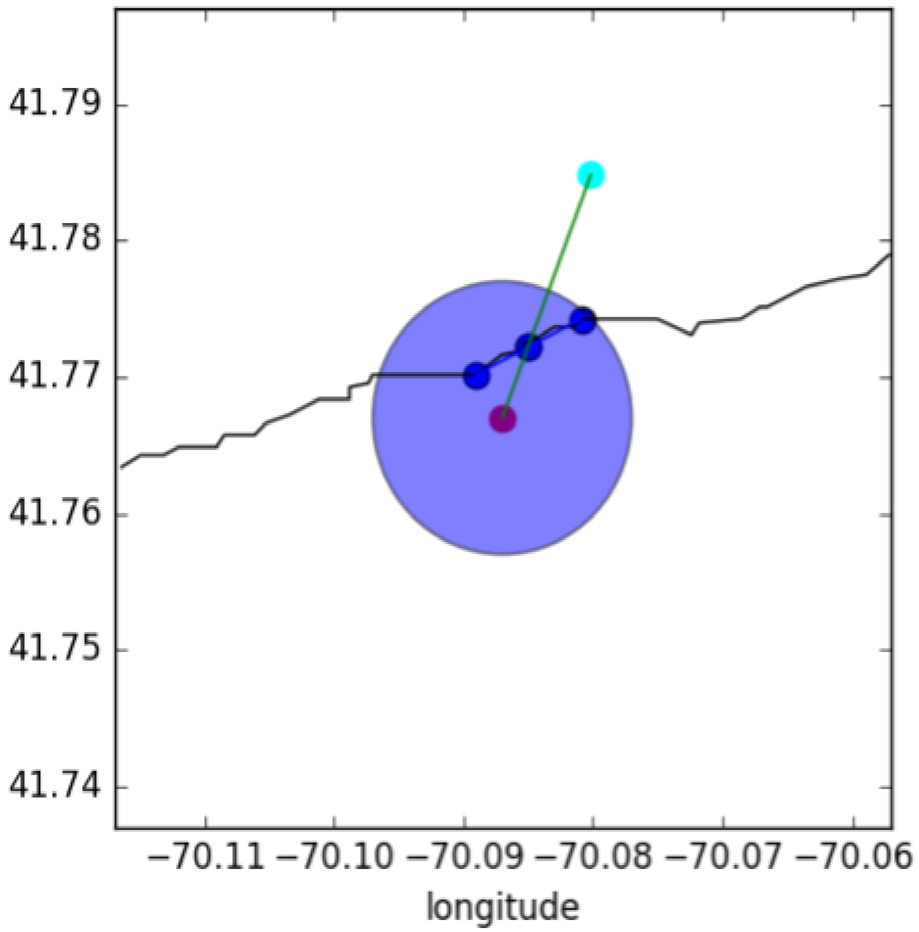

Figure S2_3. **Complex method same as in Figure S2_2 but with the radius of the circle is 1.1km.**
