# Supplementary material for: On simulating cold-stunned sea turtle strandings on Cape Cod, Massachusetts
Source: PLoS One. 2019 Dec 4;14(12):e0204717. doi: 10.1371/journal.pone.0204717 (PMC6892539; doi:10.1371/journal.pone.0204717)
Supplement: S3 Supplementary material CCBay manuscript — This file includes two sections on 1) animating tracks including links to the code and example animations and 2) a description of the drifter archive including three figures with some details on the years and months drifter tracks are available for Cape Cod Bay as well as the total on the entire Northeast Shelf. (PDF) [file pone.0204717.s003.pdf]

Supplementary Material related to:

**"On simulating cold stunned turtle strandings on Cape Cod"**

# Appendix 3: More on drifters

## On animating tracks and wind with Python

In order to best visualize the variability of circulation in Cape Cod Bay, we animated the drifter tracks and overlaid a representation of wind in the form of a web-served gif file. The code for this animation can be found here. It uses two input files, the ascii data for a particular batch of drifters tracks and a time series of NCEP wind for a nearby grid point. The drifter data can be found at NOAA's Northeast Fisheries Science Center page here and the NCEP wind was downloaded from their FTP site. After running the Python code "animate_drifter_Basemap.py" we have a set of ordered frame*.png files in a particular directory where we then run the Linux "convert -delay 10 -loop 0 frame*.png /net/pubweb_html/drifter/drift_audubon_2018_1.gif", for example. The resulting animation can be found at:

https://www.nefsc.noaa.gov/drifter/drift_audubon_2018_1.gif

## Tracks in the archive

While the animation above is for 2018 drifters, the code can be run on other years as well. In the last few years there has been about a dozen tracks within Cape Cod Bay as shown in Figure S3_1. The number of tracks within the bay by month is shown in Figure S3_2. The total tracks per year on the entire Northeast is shown in Figure S3_3.

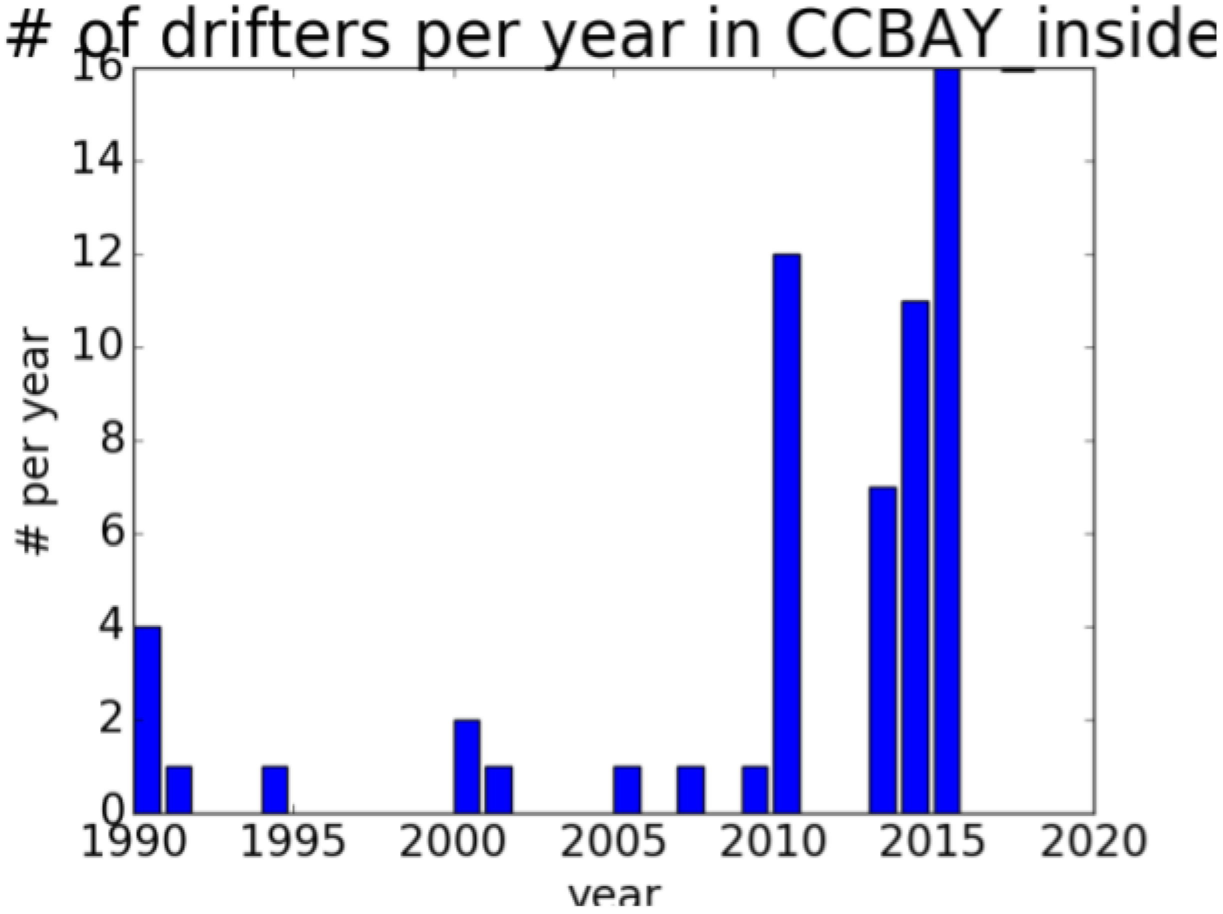


Figure S3_1. Number of drifter tracks within Cape Cod Bay prior to 2017.

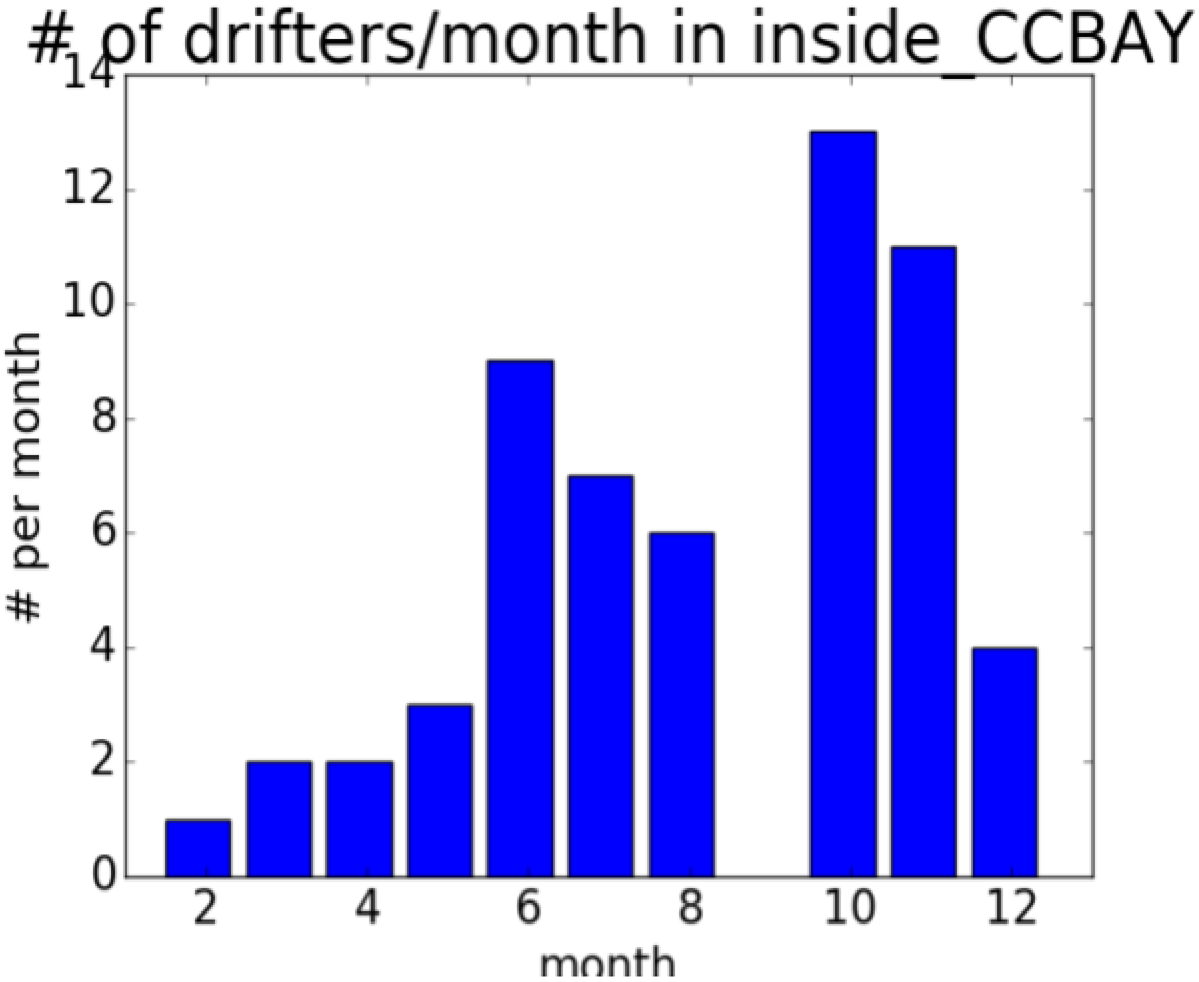

Figure S3_2. Number of drifter tracks within Cape Cod Bay per month.

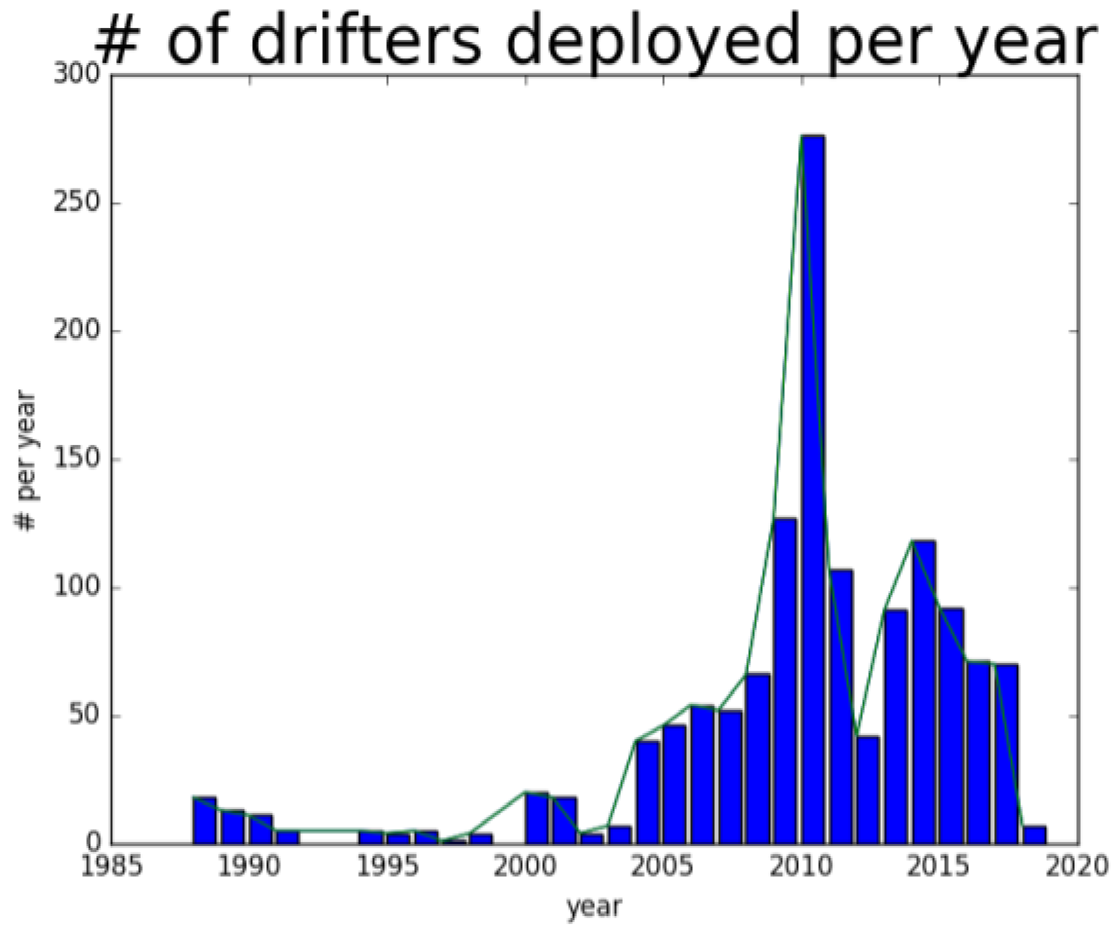


Figure S3_3. Total number of drifters deployments on Northeast Shelf by year.
